# Concordance of Prebiopsy and Postbiopsy Diagnosis in Hospitalized Patients with Acute Kidney Injury

**DOI:** 10.34067/KID.0000001151

**Published:** 2026-02-11

**Authors:** Maxine McGredy, David Hu, Heather Thiessen Philbrook, Celia P. Corona-Villalobos, Avi Z. Rosenberg, Dennis G. Moledina, Steven G. Coca, Chirag R. Parikh, Steven Menez

**Affiliations:** 1 Department of Medicine, Johns Hopkins University School of Medicine, Baltimore, Maryland; 2 Division of Nephrology, Department of Medicine, Johns Hopkins University School of Medicine, Baltimore, Maryland; 3 Division of Renal Pathology, Department of Pathology, Johns Hopkins University School of Medicine, Baltimore, Maryland; 4 Section of Nephrology, Department of Internal Medicine, Yale University School of Medicine, New Haven, Connecticut; 5 Division of Nephrology, Department of Medicine, Icahn School of Medicine at Mount Sinai, New York, New York

**Keywords:** AKI, kidney biopsy

## Abstract

**Key Points:**

- Kidney biopsy remains the gold standard for diagnosis of kidney disease.
- Prebiopsy and postbiopsy diagnosis concordance is low for acute interstitial nephritis and acute tubular injury.

**Background:**

Percutaneous kidney biopsy remains the gold standard for evaluation of AKI, given that clinicians' prebiopsy clinical impression of AKI, on the basis of history, examination, and noninvasive testing may not lead to a unifying diagnosis. In this study, we evaluated the concordance of prebiopsy clinical diagnosis with postbiopsy final diagnosis among patients with clinical AKI.

**Methods:**

We leveraged data collected prospectively through the Novel Approaches in the Investigation of Kidney Disease Study between 2020 and 2023, in which adult patients admitted to the Johns Hopkins Hospital and scheduled for clinical kidney biopsies consented to provide biosamples paired with data collection. Up to three prebiopsy clinical diagnoses were recorded for each patient, along with up to three postbiopsy diagnoses, adjudicated by a study nephrologist postbiopsy. We investigated the concordance of prebiopsy and postbiopsy diagnoses among patients with suspected acute interstitial nephritis (AIN) or acute tubular injury (ATI).

**Results:**

Among 164 total participants, 29 patients had a suspected clinical diagnosis of AIN and 47 had a suspected clinical diagnosis of ATI prebiopsy. Among the participants with suspected AIN, only seven (24%) had AIN confirmed on biopsy. Of the 22 biopsies without AIN present, alternative diagnoses noted on histology included FSGS, ATI, and various glomerular diseases. Of 47 participants with suspected ATI, ATI was confirmed on biopsy in 27 (57%) participants. In the 20 biopsies without ATI present, alternative histological findings also included glomerular diseases, diabetic nephropathy, and FSGS predominantly.

**Conclusions:**

Patients with suspected ATI or AIN who undergo percutaneous kidney biopsy are often found to have alternative, significant findings present on histology. Among patients without relative or absolute contraindications, kidney biopsy remains an essential part of clinical evaluation, while future research should focus on the development of noninvasive approaches for kidney disease diagnosis.

## Visual Abstract

**Figure:**
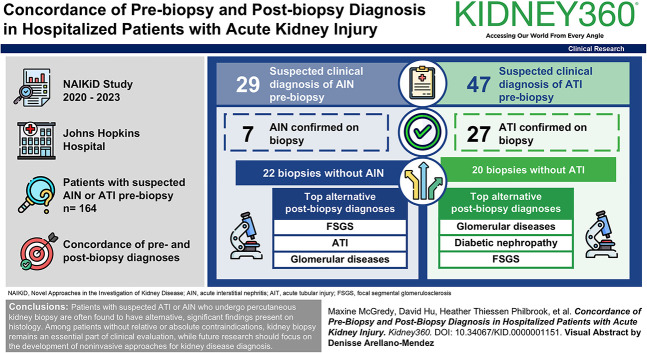

## Introduction

Kidney biopsy remains the gold standard for determining diagnosis and prognosis in kidney disease, despite increased research investigating blood and urine biomarkers which has improved precision in understanding both AKI and CKD.^1–4^ Up to 20% of patients during any given hospitalization may present with or develop an AKI.^5^ The 2012 Kidney Disease Improving Global Outcomes guidelines for diagnosis of AKI recommend consideration of kidney biopsy in patients with severe AKI in whom etiology remains unclear.^6^ However, kidney biopsy remains an invasive procedure with risks including hematoma development (11%), transfusion requirement (1.3%), and rarely nephrectomy and death, with higher rates in hospitalized patients.^7^ Risks of biopsy must be balanced with the need for an accurate diagnosis of AKI etiology.

Patients with suspected acute tubular injury (ATI) or acute interstitial nephritis (AIN) may not undergo kidney biopsy in certain scenarios, including when clinical suspicion strongly favors a specific diagnosis, or when procedural risks including postbiopsy bleeding complications outweigh the potential benefit because of safety concerns.^8^ However, prior research has demonstrated that concordance of prebiopsy clinical diagnosis with postbiopsy final diagnosis among patients with AKI remains poor.^9^ The accurate diagnosis of patients with either AIN or ATI is essential because patient management may be significantly affected, including the discontinuation of medications and the initiation of steroids for patients with AIN. Furthermore, a misdiagnosis can lead to inappropriate therapy, continuation of nephrotoxic medications, delayed AKI recovery, or permanent kidney function decline. In this study, we sought to understand the concordance between prebiopsy clinical diagnoses and postbiopsy final diagnoses among patients with suspected AIN or ATI and how biopsy results changed management.

## Methods

Our cohort included adult patients admitted to the Johns Hopkins Hospital (JHH) who underwent native kidney biopsies between September 1, 2020, and December 31, 2023, and were enrolled in the Novel Approaches in the Investigation of Kidney Diseases (NAIKiD) Study. The NAIKiD Study was initiated in September 2020 with the goal of enrolling both inpatients and outpatients scheduled for clinically indicated native kidney biopsies at the JHH to contribute blood, urine, and kidney tissue toward building a kidney biorepository. Patients were approached to be included in the NAIKiD study after they had already been determined to require kidney biopsy by primary nephrology team and after the patient had already been consented for biopsy. Study exclusion criteria included 18 years or older and inability to provide written informed consent. In addition to active data collection through patient questionnaires taken at the time of study enrollment, patients enrolled into the NAIKiD Study were asked to consent to passive data collection within the Johns Hopkins Epic electronic medical record system, extracted through the Johns Hopkins Kidney Precision Medicine Center of Excellence (see Supplemental Methods). Enrolled patients were additionally approached for consent allowing external electronic medical record data extraction through the Chesapeake Regional Information System for Our Patients regional health information exchange.

For research kidney tissue collection, patients were asked to consent to an extra core of kidney tissue, unless they met the following exclusion criteria: hemoglobin level <8 g/dl, platelet count <75,000, use of aspirin within 5 days prior biopsy, use of nonsteroidal anti-inflammatory drugs within 48 hours before biopsy, confirmed or suspected pregnancy, inability to withdraw systemic anticoagulation at least 24 hours before or 48 hours after biopsy, international normalized ratio >1.4, severe iodine allergy, Jehovah's Witness or otherwise unwilling to receive a blood transfusion, or any other concerns by the biopsy operator where an extra core of kidney tissue cannot be safely obtained. For patients who did not consent to, or were not eligible for, an extra core of kidney tissue, residual tissue was obtained after clinical processing by the division of kidney pathology. Patients with hemoglobin <7 g/dl were excluded from consideration for blood sample collection.

The top three prebiopsy clinical diagnoses and top three final postbiopsy diagnoses for each participant were determined by a senior study nephrologist. Prebiopsy clinical diagnoses were adjudicated by the study nephrologist on the basis of all available clinical nephrology notes up until the time of biopsy, while postbiopsy diagnoses were determined on the basis of the findings recorded from the clinical pathology report as well as any additional laboratory or clinical data available at the time of final adjudication. Table 1 details a full list of prebiopsy clinical diagnoses and postbiopsy final diagnoses.

**Table 1:** Clinical and final diagnoses in the novel approaches in the investigation of kidney disease study

Prebiopsy Clinical Diagnoses	Postbiopsy Final Diagnoses
AIN	AIN
ATI	ATI
Amyloid, fibrillary, or immunotactoid GN	Alport disease
Anti-GBM disease	Amyloid
C3 GN, dense deposit disease	Anti-GBM disease
Chronic interstitial nephritis	Autosomal dominant tubulointerstitial disease
Crystalline nephropathy	C3 GN
Diabetic nephropathy	Chronic interstitial nephritis
Fabry disease	Contrast associated nephropathy
FSGS	Crystalline nephropathy
HIVAN	Cystic kidney disease (other than PKD)
Hypertension-attributed glomerulosclerosis, arteriosclerosis	Dense deposit disease
IgA nephropathy	Diabetic nephropathy
IgG4-related disease	Fabry disease
Membranous nephropathy	Fibrillary arteritis
Minimal change disease	FSGS
Monoclonal gammopathy	Global glomerulosclerosis
Pauci-immune GN	HIVAN
Peri/post-infectious GN	Hyperoxaluria, primary
SLE nephritis	Hyperoxaluria, secondary
Thrombotic microangiopathy	HTN-attributed glomerulosclerosis, arteriosclerosis
NSAID nephropathy	IgA nephropathy
Pre-renal azotemia	IgG4-related disease
Scleroderma renal crisis	Immunotactoid arteritis
Contrast nephropathy	Light chain or heavy chain deposition disease
Cryoglobulinemic vasculitis	Membrano-proliferative GN pattern
Tubular atrophy, interstitial fibrosis	Membranous nephropathy, primary
Thin basement membrane disease	Membranous nephropathy, secondary
Other	Minimal change disease, primary
	Minimal change disease, secondary
	MGRS
	Myeloma cast nephropathy
	Pauci-immune GN
	Peri/postinfectious GN
	Phosphate nephropathy, acute
	SLE nephritis
	Thin basement membrane disease
	Thrombotic microangiopathy
	Urate nephropathy, acute
	Tubular atrophy, interstitial fibrosis
	Normal/unremarkable
	Cryoglobulinemic vasculitis
	Other

AIN, acute interstitial nephritis; ATI, acute tubular injury; GBM, glomerular basement membrane; HIVAN, HIV-associated nephropathy; HTN, hypertension; MGRS, monoclonal gammopathy of renal significance; NSAID, nonsteroidal anti-inflammatory drug; PKD, polycystic kidney disease.

For this study, baseline serum creatinine was determined, and the associated baseline eGFR was calculated, on the basis of the median of all prior outpatient serum creatinine measurements in the year before hospitalization in which the kidney biopsy was performed, assuming at least three outpatient values. If only two serum creatinine values were available, the mean of these values was used. If only one serum creatinine was available, that single value was used. If no available outpatient serum creatinine values were available, the lowest serum creatinine during hospitalization was taken (before the start of any hemodialysis) to determine baseline kidney function.

## Results

A total of 164 participants consented to the NAIKiD Study and underwent inpatient native kidney biopsy at JHH between September 1, 2020, and December 31, 2023. Mean participant age was 52.6 (±16.1) years, 48% of participants were female, and 45% were of Black race. Baseline eGFR before hospitalization was 58.9 ml/min per 1.73 m^2^, with peak serum creatinine of 4.6 mg/dl during hospitalization (Table 2).

**Table 2:** Patient characteristics

Variable	Overall^a^ (*N*=164)
**At the time of biopsy**	
Age	52.6 (16.1)
Gender: Female	78 (48%)
Race	
*Black*	74 (45%)
*Other*	19 (12%)
*White*	71 (43%)
Ethnicity: Hispanic	12 (7%)
History of hypertension	79 (48%)
History of diabetes mellitus	44 (27%)
BMI at time of biopsy (kg/m^2^)	27.8 (7.06)
Hospital floor/location	
*Floor*	105 (64%)
*Intermediate care*	50 (31%)
*Intensive care unit*	9 (6%)
*Peak serum creatinine during hospitalization (mg/dl)*	4.55 (3.4)
KDIGO AKI stage	
*No AKI*	61 (37%)
*Stage 1*	28 (17%)
*Stage 2*	31 (19%)
*Stage 3*	44 (27%)
**Baseline** ^b^	
Baseline eGFR^c^	58.9 (33)
Baseline serum creatinine (mg/dl)	1.77 (1.21)

BMI, body mass index; KDIGO, Kidney Disease Improving Global Outcomes.

a Variables reported in mean (SD) or *n* (%).

b Baseline kidney function was determined by outpatient serum creatinine measurements in the year prior to hospitalization in which the kidney biopsy was performed.

c By CKD Epidemiology Collaboration 2021 equation.

Of the 164 participants, AIN was clinically suspected in 29 participants prebiopsy, with only seven showing evidence of AIN on biopsy. A total of 47 of the 164 participants had clinically suspected ATI prebiopsy. This was confirmed on biopsy in 27 participants. In total, sensitivity for the diagnosis of AIN and ATI was 41% and 44%, respectively, while specificity was 85% and 81%, respectively (Supplemental Figure 1). Supplemental Figure 1C summarizes these measures of diagnostic performance. In addition, positive predictive value (PPV) for diagnosis of AIN was 24.1%; 95% confidence interval, 12.2% to 42.1%. For ATI, PPV was 57.4%; 95% confidence interval, 43.3% to 70.5%.

**Figure 1:**
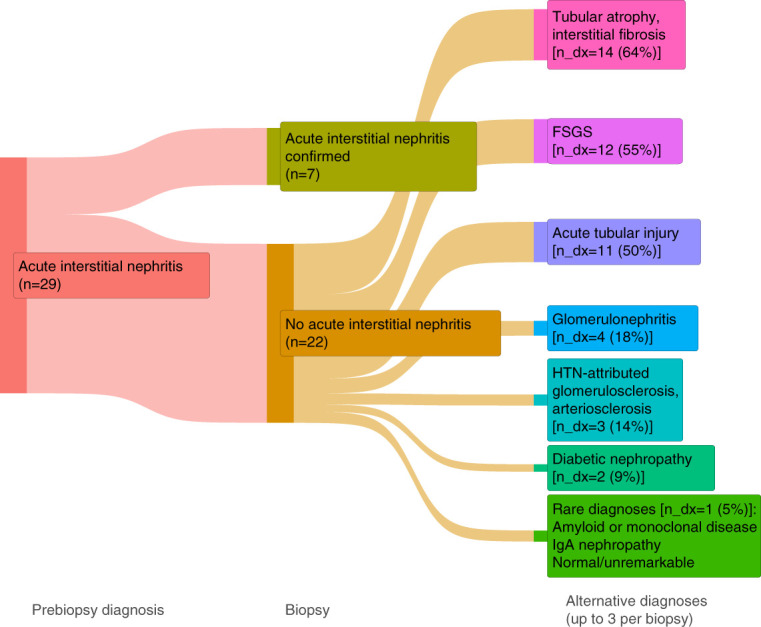
**Flow diagram demonstrating final adjudicated diagnoses among participants with suspected diagnosis of AIN before kidney biopsy.** The first section (labeled Prebiopsy diagnosis) reflects the number of biopsies (n) in which AIN was suspected, before biopsy. The second section (labeled Biopsy) highlights the number of biopsies in which suspected AIN was confirmed (seven of 29). The third section (labeled Alternative diagnoses) reflects the other diagnoses (n_dx, up to three recorded per biopsy) that were seen among the 22 biopsies in which suspected AIN was ruled out. AIN, acute interstitial nephritis; HTN, hypertension.

**Figure 2:**
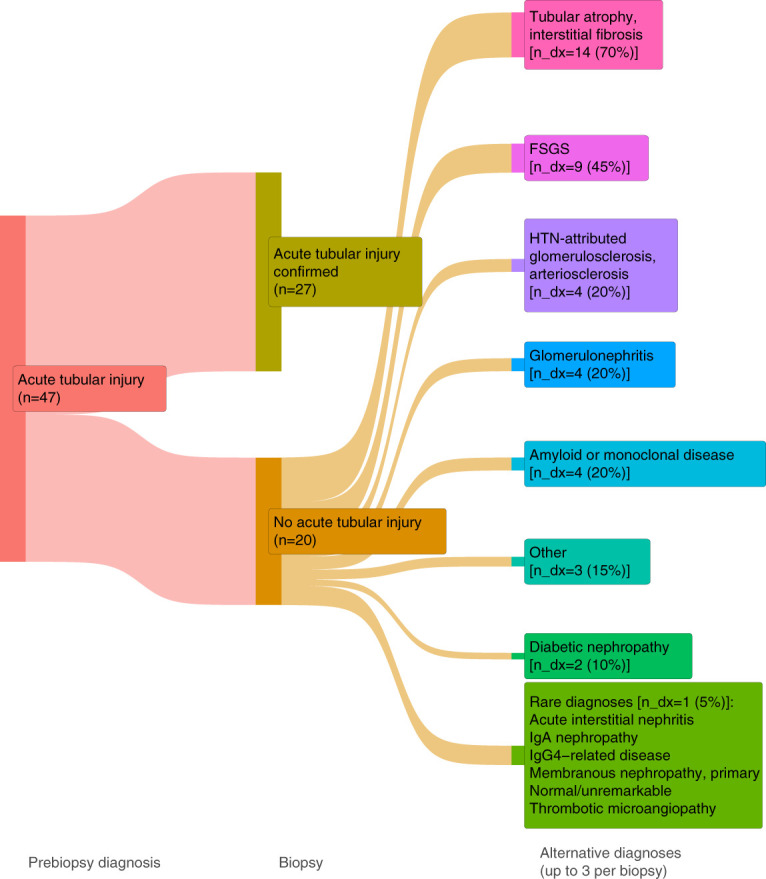
**Flow diagram demonstrating final adjudicated diagnoses among participants with suspected diagnosis of ATI prior to kidney biopsy.** The first section (labeled Prebiopsy diagnosis) reflects the number of biopsies (n) in which ATI was suspected, before biopsy. The second section (labeled Biopsy) highlights the number of biopsies in which suspected ATI was confirmed (27 of 47). The third section (labeled Alternative diagnoses) reflects the other diagnoses (n_dx, up to three recorded per biopsy) that were seen among the 20 biopsies in which suspected ATI was ruled out. ATI, acute tubular injury.

Of the 22 patients without AIN diagnosed on biopsy, multiple histological findings were identified, including FSGS, ATI, and glomerular diseases (Figure 1). AIN was not suspected clinically in 135 patients but was identified on biopsy in ten patients (negative predictive value [NPV]=93%). In the 20 participants without ATI on biopsy, alternative findings included glomerular diseases, diabetic nephropathy, and FSGS (Figure 2). ATI was not suspected clinically in 117 patients, but was confirmed on biopsy in 34 patients (NPV=71%).

We evaluated cases in which neither AIN nor ATI were suspected prebiopsy but were noted on histology. Of 135 participants without suspected AIN prebiopsy, ten of 135 participants (7.4%) unexpectedly had histologic AIN (Supplemental Table 1). Of these, seven underwent a change in management because of biopsy results; six patients were initiated on steroids, and medications were discontinued for four patients. Of these four, medications that were discontinued included immune checkpoint inhibitors (ICI), antibiotics, proton pump inhibitors, and BP medications. Similarly, of the 117 participants in whom ATI was not suspected prebiopsy, 34 had histologically confirmed ATI (29%). Supplemental Figure 2 highlights the frequency of individual pathological findings identified across the 164 biopsied patients.

## Discussion

In this study, we found that clinically suspected AIN was confirmed on biopsy in only a quarter of participants, while suspected ATI was confirmed in just over half of patients. Clinically unsuspected AIN was discovered on biopsy in only a small number of patients, with a clear effect on patient management. We demonstrated that clinical diagnosis of AKI differs from final histologic diagnosis in the majority of cases, with potential management changes.

In contrast to prior studies, our study showed a low rate of diagnosis concordance. Haas and colleagues previously assessed utility of kidney biopsy and found agreement among clinical and pathological diagnoses in 67% of cases.^10^ However, in that study, kidney biopsies were typically pursued among patients with greater diagnostic uncertainty, primarily in patients with rapidly progressive glomerulonephritides. For glomerular diseases in particular, plasma-based serological testing has become widely available, increasing accuracy of clinical diagnosis.^11^ Noninvasive models to predict AIN likelihood have been recently developed, yet clinical utilization remains limited.^9,12^ Indeed, since the development and increasing utilization of ICI therapy, the accurate diagnosis of ICI-associated AIN has had significant implications for cancer treatment and subsequent mortality. In a recent case series, Rashidi *et al*. described specific instances in which patients receiving ICI therapy suffered AKI with a high clinical suspicion of AIN, but with kidney biopsy revealing features of ATI alone. Clarifying ATI versus ICI-associated AIN informed the decision to continue immunotherapy and prevented the need to expose patients to high-dose steroids.^13^

This study has several key strengths. We systematically compared prebiopsy clinical diagnoses with final diagnoses, through a comprehensive review of clinical data including clinical notes, laboratory data, and pathological reporting by a study nephrologist. Importantly, we demonstrated how the diagnosis of AIN on biopsy, when not clinically suspected prior to biopsy, significantly impacted postbiopsy management, underscoring the clinical utility of kidney biopsy in accurate diagnosis and subsequent clinical care. Future investigations can further explore how biopsy-proven diagnoses, especially glomerulonephritides not anticipated before biopsy, affect therapeutic decision-making and patient management.

This study has important limitations. This was a single-center study with a small sample size, limited to evaluating diagnosis concordance in suspected AIN or ATI. Final diagnoses were determined based on findings recorded on the clinical pathology report, without additional pathologist adjudication. While we indicated management changes among patients with clinically unsuspected AIN, we did not perform a similar analysis for ATI given the heterogeneity in ATI management, and the difficulty in determining whether a biopsy-confirmed diagnosis of ATI, when not suspected prebiopsy, directly influenced subsequent clinical care. We believe this study warrants further investigation into how biopsy changed management in patients who were newly found to have ATI, but this was outside the scope of our original study. This study only included patients who were deemed by the primary nephrology team to require biopsy for diagnosis of the etiology of AKI, thereby excluding patients with clear cut clinical diagnoses. In the case of clear clinical diagnosis, biopsy would very likely not have been pursued. As a result, the true accuracy of clinical diagnosis may be much higher than what is reported in this study. In addition, while in practice many differential diagnoses are considered when pursuing kidney biopsy, in this study, we only assessed the top three differentials listed in clinical records to calculate PPV/NPV. Furthermore, this study did not assess longitudinal follow-up on clinical outcomes after diagnosis or change in management. Additional clinical phenotyping, including the presence of sepsis among patients with or without ATI, was not available for this analysis but could provide important insights to be explored in future studies.

In conclusion, we observed poor concordance between pre-biopsy clinical diagnoses of AIN and ATI with final adjudicated diagnoses. Kidney biopsy remains an essential tool for the accurate diagnosis of AKI at the present, and future work should explore the development and validation of noninvasive approaches for kidney disease diagnosis.

## Supplementary Material

## Data Availability

Original data generated for the study will be made available upon reasonable request to the corresponding author. Data Type: Raw Data/Source Data. Reason for Restricted Access: Per protocol.

## Declarative Statements

This study includes clinical experimentation and complies with the Declaration of Helsinki. The clinical and research activities being reported are consistent with the Principles of the Declaration of Istanbul as outlined in the Declaration of Istanbul on Organ Trafficking and Transplant Tourism.

## Supplemental Material

Supplemental Figure 1. Confusion matrices and diagnostic performance of prebiopsy clinical and post biopsy final diagnoses.

Supplemental Figure 2. Spectrum and frequency of pathological findings among the 164 patients who underwent kidney biopsy.

Supplemental Table 1. Clinical and final diagnoses in the NAIKID study.

Supplemental Table 2. Baseline patient characteristics

Supplemental Table 3. Change in management among participants without suspected prebiopsy AIN but confirmed on biopsy.
