# Supplementary material for: Concordance of Prebiopsy and Postbiopsy Diagnosis in Hospitalized Patients with Acute Kidney Injury
Source: Kidney360. 2026 Feb 11;7(5):1027–33. doi: 10.34067/KID.0000001151 (PMC13229441; doi:10.34067/KID.0000001151)
Supplement: Supplementary file 1 [file kidney360-7-1027-s001.pdf]

ASN American Society of Nephrology | JASN Journal of the American Society of Nephrology | KIDNEY360 Accessing Our World From Every Angle | CJASN Clinical Journal of the American Society of Nephrology

ASN Journal Disclosure Form

As per ASN journal policy, I have disclosed any financial relationships or commitments I have held in the past 36 months as included below. I have listed my Current Employer below to indicate there is a relationship requiring disclosure. If no relationship exists, my Current Employer is not listed.

S. Coca reports the following:
Employer: Icahn School of Medicine at Mount Sinai; Mount Sinai owns part of Renalytix; Consultancy: Renalytix, Bayer, Alexion, SC Pharma, Whiteswell, Vera Therapeutics, Nuwellis; Ownership Interest: Renalytix; Research Funding: Renalytix, ProKidney; Patents or Royalties: Renalytix; and Other Interests or Relationships: Associate Editor for Kidney360, Editorial Boards of JASN, CJASN, Kidney International.

I understand that the information above will be published within the journal article, if accepted, and that failure to comply and/or to accurately and completely report the potential financial conflicts of interest could lead to the following: 1) Prior to publication, article rejection, or 2) Post-publication, sanctions ranging from, but not limited to, issuing a correction, reporting the inaccurate information to the authors' institution, banning authors from submitting work to ASN journals for varying lengths of time, and/or retraction of the published work.

Name: Steven G. Coca
Manuscript ID: K360-2025-001058R2
Manuscript Title: Concordance of pre-biopsy and post-biopsy diagnosis in hospitalized patients with acute kidney injury
Date of Completion: January 14, 2026
Disclosure Updated Date: October 3, 2025

ASN
American Society of Nephrology
JASN
Journal of the American Society of Nephrology
KIDNEY360
Accessing Our World From Every Angle
CJASN
Clinical Journal of the American Society of Nephrology

ASN Journal Disclosure Form

As per ASN journal policy, I have disclosed any financial relationships or commitments I have held in the past 36 months as included below. I have listed my Current Employer below to indicate there is a relationship requiring disclosure. If no relationship exists, my Current Employer is not listed.

C. Corona Villalobos reports the following:
Employer: Johns Hopkins University; and Research Funding: Bioporto Diagnostics.

I understand that the information above will be published within the journal article, if accepted, and that failure to comply and/or to accurately and completely report the potential financial conflicts of interest could lead to the following: 1) Prior to publication, article rejection, or 2) Post-publication, sanctions ranging from, but not limited to, issuing a correction, reporting the inaccurate information to the authors' institution, banning authors from submitting work to ASN journals for varying lengths of time, and/or retraction of the published work.

Name: Celia Pamela Corona Villalobos
Manuscript ID: K360-2025-001058R2
Manuscript Title: Concordance of pre-biopsy and post-biopsy diagnosis in hospitalized patients with acute kidney injury
Date of Completion: January 7, 2026
Disclosure Updated Date: January 7, 2026

ASN American Society of Nephrology
JASN Journal of the American Society of Nephrology
KIDNEY360 Accessing Our World From Every Angle
CJASN Clinical Journal of the American Society of Nephrology

ASN Journal Disclosure Form

As per ASN journal policy, I have disclosed any financial relationships or commitments I have held in the past 36 months as included below. I have listed my Current Employer below to indicate there is a relationship requiring disclosure. If no relationship exists, my Current Employer is not listed.

D. Hu reports the following:
Employer: Johns Hopkins University

I understand that the information above will be published within the journal article, if accepted, and that failure to comply and/or to accurately and completely report the potential financial conflicts of interest could lead to the following: 1) Prior to publication, article rejection, or 2) Post-publication, sanctions ranging from, but not limited to, issuing a correction, reporting the inaccurate information to the authors' institution, banning authors from submitting work to ASN journals for varying lengths of time, and/or retraction of the published work.

Name: David Hu
Manuscript ID: K360-2025-001058R2
Manuscript Title: Concordance of pre-biopsy and post-biopsy diagnosis in hospitalized patients with acute kidney injury
Date of Completion: January 14, 2026
Disclosure Updated Date: January 14, 2026

ASN American Society of Nephrology · JASN Journal of the American Society of Nephrology · KIDNEY360 Accessing Our World From Every Angle · CJASN Clinical Journal of the American Society of Nephrology

ASN Journal Disclosure Form

As per ASN journal policy, I have disclosed any financial relationships or commitments I have held in the past 36 months as included below. I have listed my Current Employer below to indicate there is a relationship requiring disclosure. If no relationship exists, my Current Employer is not listed.

M. McGredy has nothing to disclose.

I understand that the information above will be published within the journal article, if accepted, and that failure to comply and/or to accurately and completely report the potential financial conflicts of interest could lead to the following: 1) Prior to publication, article rejection, or 2) Post-publication, sanctions ranging from, but not limited to, issuing a correction, reporting the inaccurate information to the authors' institution, banning authors from submitting work to ASN journals for varying lengths of time, and/or retraction of the published work.

Name: Maxine McGredy
Manuscript ID: K360-2025-001058R2
Manuscript Title: Concordance of pre-biopsy and post-biopsy diagnosis in hospitalized patients with acute kidney injury
Date of Completion: January 5, 2026
Disclosure Updated Date: January 5, 2026

ASN
American Society of Nephrology
JASN
Journal of the American Society of Nephrology
KIDNEY360
Accessing Our World From Every Angle
CJASN
Clinical Journal of the American Society of Nephrology

ASN Journal Disclosure Form

As per ASN journal policy, I have disclosed any financial relationships or commitments I have held in the past 36 months as included below. I have listed my Current Employer below to indicate there is a relationship requiring disclosure. If no relationship exists, my Current Employer is not listed.

S. Menez reports the following:
Employer: Johns Hopkins University School of Medicine; Consultancy: Veracity Health; and Patents or Royalties: McGraw Hill.

I understand that the information above will be published within the journal article, if accepted, and that failure to comply and/or to accurately and completely report the potential financial conflicts of interest could lead to the following: 1) Prior to publication, article rejection, or 2) Post-publication, sanctions ranging from, but not limited to, issuing a correction, reporting the inaccurate information to the authors' institution, banning authors from submitting work to ASN journals for varying lengths of time, and/or retraction of the published work.

Name: Steven Menez
Manuscript ID: K360-2025-001058R2
Manuscript Title: Concordance of pre-biopsy and post-biopsy diagnosis in hospitalized patients with acute kidney injury
Date of Completion: January 6, 2026
Disclosure Updated Date: January 6, 2026

ASN
American Society of Nephrology
JASN
Journal of the American Society of Nephrology
KIDNEY360
Accessing Our World From Every Angle
CJASN
Clinical Journal of the American Society of Nephrology

ASN Journal Disclosure Form

As per ASN journal policy, I have disclosed any financial relationships or commitments I have held in the past 36 months as included below. I have listed my Current Employer below to indicate there is a relationship requiring disclosure. If no relationship exists, my Current Employer is not listed.

D. Moledina reports the following:
Employer: Yale University School of Medicine; Consultancy: BioHaven, Inc.; Ownership Interest: Predict AIN, LLC; CT scientific, LLC; Research Funding: NIDDK; Honoraria: Healthcentral; Patents or Royalties: DGM is a coinventor of the pending patent application "Methods and Systems for Diagnosis of Acute Interstitial Nephritis"; and Advisory or Leadership Role: ASN journals, editorial board member; Evidence to action: the official journal of MDCalc, editorial board member.

I understand that the information above will be published within the journal article, if accepted, and that failure to comply and/or to accurately and completely report the potential financial conflicts of interest could lead to the following: 1) Prior to publication, article rejection, or 2) Post-publication, sanctions ranging from, but not limited to, issuing a correction, reporting the inaccurate information to the authors' institution, banning authors from submitting work to ASN journals for varying lengths of time, and/or retraction of the published work.

Name: Dennis G. Moledina
Manuscript ID: K360-2025-001058R2
Manuscript Title: Concordance of pre-biopsy and post-biopsy diagnosis in hospitalized patients with acute kidney injury
Date of Completion: January 7, 2026
Disclosure Updated Date: January 7, 2026

ASN
American Society of Nephrology
JASN
Journal of the American Society of Nephrology
KIDNEY360
Accessing Our World From Every Angle
CJASN
Clinical Journal of the American Society of Nephrology

ASN Journal Disclosure Form

As per ASN journal policy, I have disclosed any financial relationships or commitments I have held in the past 36 months as included below. I have listed my Current Employer below to indicate there is a relationship requiring disclosure. If no relationship exists, my Current Employer is not listed.

C. Parikh reports the following:
Employer: Johns Hopkins University School of Medicine; Ownership Interest: Renalytix; Research Funding: National Institute of Diabetes and Digestive and Kidney Diseases (NIDDK); National Heart, Lung and Blood Institute (NHLBI); AstraZeneca; Alexion; and Advisory or Leadership Role: AstraZeneca; Alexion; Panoramic Science.

I understand that the information above will be published within the journal article, if accepted, and that failure to comply and/or to accurately and completely report the potential financial conflicts of interest could lead to the following: 1) Prior to publication, article rejection, or 2) Post-publication, sanctions ranging from, but not limited to, issuing a correction, reporting the inaccurate information to the authors' institution, banning authors from submitting work to ASN journals for varying lengths of time, and/or retraction of the published work.

Name: Chirag R. Parikh
Manuscript ID: K360-2025-001058R2
Manuscript Title: Concordance of pre-biopsy and post-biopsy diagnosis in hospitalized patients with acute kidney injury
Date of Completion: January 15, 2026
Disclosure Updated Date: April 23, 2025

ASN American Society of Nephrology | JASN Journal of the American Society of Nephrology | KIDNEY360 Accessing Our World From Every Angle | CJASN Clinical Journal of the American Society of Nephrology

ASN Journal Disclosure Form

As per ASN journal policy, I have disclosed any financial relationships or commitments I have held in the past 36 months as included below. I have listed my Current Employer below to indicate there is a relationship requiring disclosure. If no relationship exists, my Current Employer is not listed.

A. Rosenberg reports the following:
Employer: Johns Hopkins University; and Consultancy: Nanogene.

I understand that the information above will be published within the journal article, if accepted, and that failure to comply and/or to accurately and completely report the potential financial conflicts of interest could lead to the following: 1) Prior to publication, article rejection, or 2) Post-publication, sanctions ranging from, but not limited to, issuing a correction, reporting the inaccurate information to the authors' institution, banning authors from submitting work to ASN journals for varying lengths of time, and/or retraction of the published work.

Name: Avi Z. Rosenberg
Manuscript ID: K360-2025-001058R2
Manuscript Title: Concordance of pre-biopsy and post-biopsy diagnosis in hospitalized patients with acute kidney injury
Date of Completion: January 25, 2026
Disclosure Updated Date: May 19, 2025

ASN American Society of Nephrology
JASN Journal of the American Society of Nephrology
KIDNEY360 Accessing Our World From Every Angle
CJASN Clinical Journal of the American Society of Nephrology

ASN Journal Disclosure Form

As per ASN journal policy, I have disclosed any financial relationships or commitments I have held in the past 36 months as included below. I have listed my Current Employer below to indicate there is a relationship requiring disclosure. If no relationship exists, my Current Employer is not listed.

H. Thiessen Philbrook reports the following:
Employer: Johns Hopkins University; and Other Interests or Relationships: Statistical Reviewer, ASN Journals.

I understand that the information above will be published within the journal article, if accepted, and that failure to comply and/or to accurately and completely report the potential financial conflicts of interest could lead to the following: 1) Prior to publication, article rejection, or 2) Post-publication, sanctions ranging from, but not limited to, issuing a correction, reporting the inaccurate information to the authors' institution, banning authors from submitting work to ASN journals for varying lengths of time, and/or retraction of the published work.

Name: Heather Thiessen Philbrook
Manuscript ID: K360-2025-001058R2
Manuscript Title: Concordance of pre-biopsy and post-biopsy diagnosis in hospitalized patients with acute kidney injury
Date of Completion: January 6, 2026
Disclosure Updated Date: April 29, 2025
