# Supplementary material for: Concordance of Prebiopsy and Postbiopsy Diagnosis in Hospitalized Patients with Acute Kidney Injury
Source: Kidney360. 2026 Feb 11;7(5):1027–33. doi: 10.34067/KID.0000001151 (PMC13229441; doi:10.34067/KID.0000001151)
Supplement: Supplementary file 2 [file kidney360-7-1027-s002.pdf]

SUPPLEMENTAL MATERIAL

Concordance of pre-biopsy and post-biopsy diagnosis in hospitalized patients with acute kidney injury

Max McGredy, MD[1]; David Hu, MS[2]; Heather Thiessen Philbrook, MMath[2]; Celia P. Corona-Villalobos, MD, MS[2]; Avi Z. Rosenberg, MD, PhD[3]; Dennis G. Moledina, MBBS, PhD[4]; Steven G. Coca, DO, MS[5]; Chirag R. Parikh, MD, PhD[2]; Steven Menez, MD, MHS[2]

1. Department of Medicine, Johns Hopkins University School of Medicine, Baltimore, MD

2. Division of Nephrology, Department of Medicine, Johns Hopkins University School of Medicine, Baltimore, MD

3. Division of Renal Pathology, Department of Pathology, Johns Hopkins University School of Medicine, Baltimore, MD

4. Section of Nephrology, Department of Internal Medicine, Yale University School of Medicine, New Haven, CT

5. Division of Nephrology, Department of Medicine, Icahn School of Medicine at Mount Sinai, New York, NY

Supplemental Material Table of Contents

- Supplemental Methods
- Supplemental Figure 1
- Supplemental Table 1
- Supplemental Figure 2

Supplemental Methods

Johns Hopkins Kidney Precision Medicine Center of Excellence

The Johns Hopkins IRB-approved Kidney Precision Medicine Center of Excellence (KPMCOE) was established in 2020 with the goals to transform the care of patients with kidney disease, from prevention to diagnosis to treatment, and to provide diverse opportunities for clinical research. Specifically, the major research aims of the KPMCOE are to measure clinical outcomes in patients with both acute kidney injury and progressive chronic kidney disease, applying risk assessments in real-time, to improve clinical phenotyping of patients with acute kidney injury, to identify barriers to optimal clinical care and target areas for quality improvement initiatives, and to identify candidates for enrollment in prospective studies and clinical trials. The KPMCOE utilizes the Johns Hopkins Precision Medicine Analytic Platform (PMAP), with Epic data ingested on a weekly basis among all adult patients with at least one serum creatinine measurement in the Johns Hopkins Health System, who are included in the kidney registry. For data variables of interest including laboratory measurements and clinical diagnoses, raw data from the Epic data lake are compiled and refined before variable definitions are finalized. Secondary data analyses approved by the Johns Hopkins Medicine IRB can leverage kidney registry data through the KPMCOE.

A

AIN		Post-biopsy		
		Yes	No	Total
Pre-biopsy	Yes	7	22	29
	No	10	125	135
	Total	17	147	164

B

ATI		Post-biopsy		
		Yes	No	Total
Pre-biopsy	Yes	27	20	47
	No	34	83	117
	Total	61	103	164

C.

Statistical measure	AIN	ATI
Prevalence	18%	29%
Sensitivity (true positive rate)	41%	44%
Specificity (true negative rate)	85%	81%
Precision (positive predictive value)*	24%	57%
Negative predictive value*	93%	71%
False positive rate (type I error)	15%	19%
False negative rate (type II error)	59%	56%

Supplemental Figure 1. Confusion matrices and Diagnostic performance ~~for AIN (A) and ATI(B), comparing~~of pre-biopsy clinical and post-biopsy final diagnoses.

Confusion matrices for AIN (A) and ATI (B) comparing pre-biopsy clinical and post-biopsy final diagnoses.

(C) Diagnostic performance of pre-biopsy clinical diagnoses of AIN and ATI compared with post-biopsy final diagnoses.

* Positive and negative predictive values vary with the prevalence of the disease.

Supplemental Table 1. Change in management among participants without suspected pre-biopsy AIN but confirmed on biopsy

Participant	Clinical setting	Medication change	Steroid initiation
Participant 1	History of metastatic HCC on nivolumab-lenvatinib, suffering AKI on CKD G3a	Stopped PD1 inhibitor	Started steroids
Participant 2	History of polysubstance use disorder, admitted with sepsis and found to have E. faecalis endocarditis	N/A	Started steroids
Participant 3	History of prior polysubstance use disorder, HCV, admitted in septic shock with TV/AV endocarditis	Antibiotics switched	N/A
Participant 4	History of CNS lymphoma on methotrexate and rituximab	N/A	Started steroids
Participant 5	History of glioblastoma, admitted with AKI after COVID-19 vaccination	Stopped PPI	started steroids
Participant 6	History of polysubstance use disorder, admitted with abdominal pain, nausea, and vomitting	N/A	Started steroids
Participant 7	History of hypertension and opiate use disorder on methadone, admitted for AKI	Stopped HCTZ	started steroids
Participant 8	History of hypertension, diabetes mellitus, CHF admitted with volume overload	N/A	N/A
Participant 9	History of sarcoidosis, rheumatoid arthritis, steroid-induced insulin-dependent diabetes mellitus admitted with diarrhea	N/A	N/A
Participant 10	History of hypertension, admitted with leg swelling and weight gain, found to have AKI	N/A	N/A

*N/A = not applicable

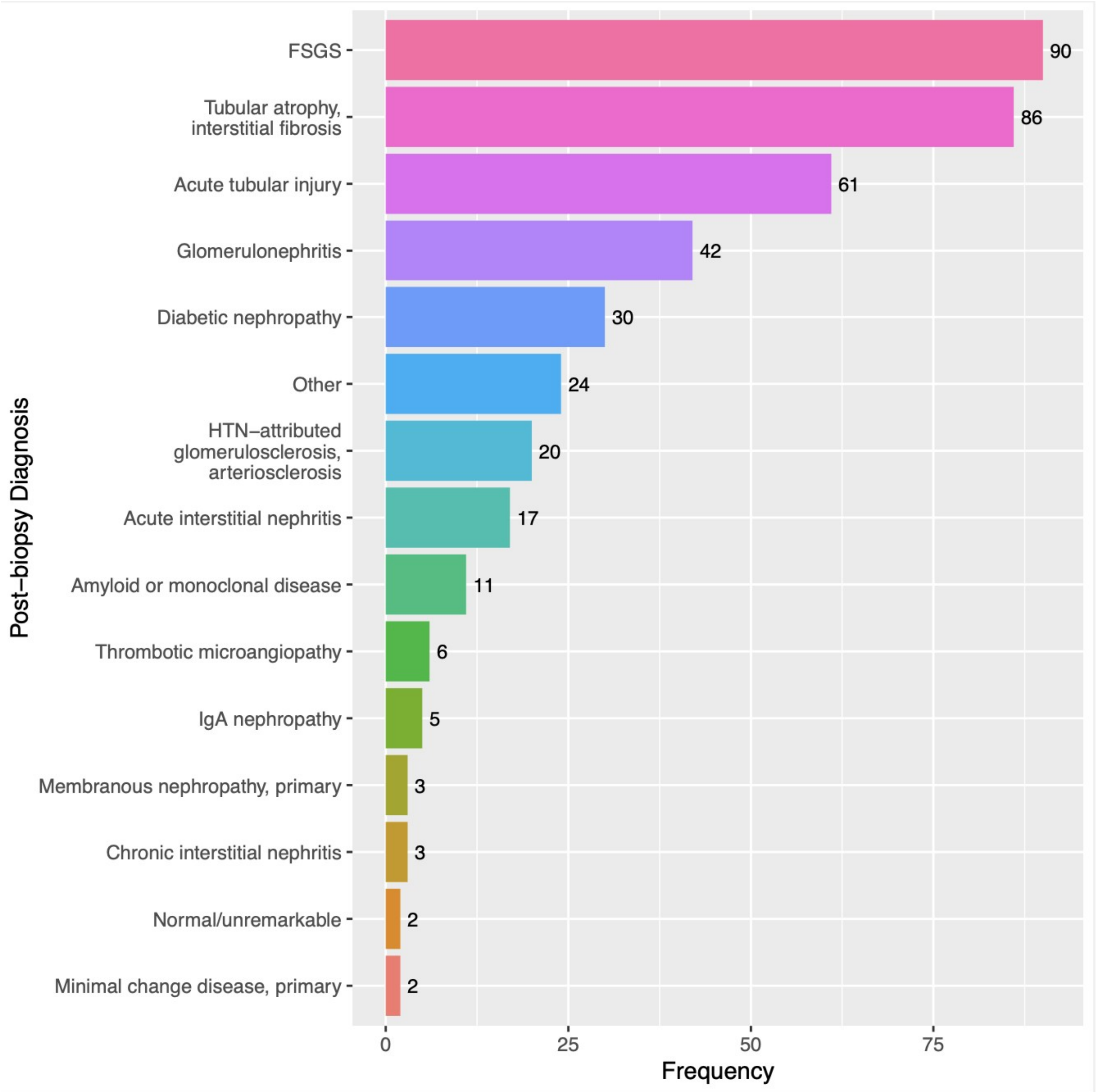

Supplemental Figure 2. spectrum and frequency of pathological findings among the 164 patients who underwent kidney biopsy.

*Up to three pathological findings could be reported on each biopsy report
